# POC1A colocalizes with POC1B in human and bovine spermatozoa centrioles

**DOI:** 10.17912/micropub.biology.002128

**Published:** 2026-08-15

**Authors:** Robert Heizelman, Yash Dixit, Derek Kluczynski, Jenna Bearden, Caitlin Stidam, Zach Madaras, Meghana Perumalla, Lydeyiah Roesner, Waleed Hamdan, Samantha Schon, Nagalakshmi Nadiminty, Tariq Shah, Puneet Sindhwani, Tomer Avidor-Reiss

**Affiliations:** 1 University of Toledo, Toledo, OH, US; 2 UMich, Ann Arbor, MI, US; 3 Department of Urology, University of Toledo, Toledo, OH, US; 4 Department of Molecular, Cellular, and Developmental Biology, University of Toledo, Toledo, OH, US

## Abstract

Infertility impacts one in seven couples globally, with one-third of cases classified as unexplained. One source of unexplained infertility is the spermatozoon centrosome, a subcellular structure composed of two remodeled centrioles (the proximal and distal centrioles) and specialized pericentriolar material, located in the spermatozoon neck. The centrosome functions as the head-neck linker in the spermatozoa. Here, we investigated the localization of the somatic cell centriole lumen protein POC1A using immunofluorescence and confocal microscopy. We found that POC1A localizes to the neck, colocalizing with POC1B, and is present in both the proximal and distal centrioles in both human and bovine spermatozoa.

**Figure 1. POC1A colocalizes with POC1B in human and bovine spermatozoa centrioles:** **A)** About 15% of couples worldwide experience infertility, and **(B)** up to a third of cases are classified as unexplained infertility. **C)** Representative image of a mammalian spermatozoon, consisting of a head, neck, and tail. **D)** Simplified diagram showing known structures of the mammalian spermatozoon neck. **E)** Confocal microscopy and **F)** HyVolution microscopy imaging results of formaldehyde-fixed human spermatozoa stained with PA5-59217 anti-POC1A antibody and H000282809-B01P anti-POC1B antibody. **G)** Confocal microscopy and **H)** HyVolution microscopy imaging results of methanol-fixed human spermatozoa stained with H00025886-B01P anti-POC1A antibody and PA5-24495 anti-POC1B antibody. **I)** Confocal microscopy and **J)** HyVolution microscopy imaging results of methanol-fixed bovine spermatozoa stained with PA5-59217 anti-POC1A antibody and H000282809-B01P anti-POC1B antibody. **K)** Confocal microscopy and **L)** HyVolution microscopy imaging results of methanol-fixed bovine spermatozoa stained with H00025886-B01P anti-POC1A antibody and PA5-24495 anti-POC1B antibody. **M-N)** Summary of the observed localization of POC1A and POC1B at the proximal centriole and distal centriole in both human ( **M** ) and bovine ( **N** ) species. **O** ) POC1A and POC1B DC:PC ratios in human and bovine sperm. P, PA5-59217 anti-POC1A/H000282809-B01 anti-POC1B antibody pair; H, H00025886-B01P anti-POC1A/PA5-24495 anti-POC1B antibody pair; Co, confocal imaging; Hy, HyVolution imaging; HS, *Homo sapiens* ; and BT, *Bos taurus* . Error bars represent standard deviation. Asterisks indicate significant differences between POC1A and POC1B within the same condition by T-test with a p-value <0.05. Nu, nucleus; Ne, neck; Ta, tail; DC, distal centriole; PC, proximal centriole.

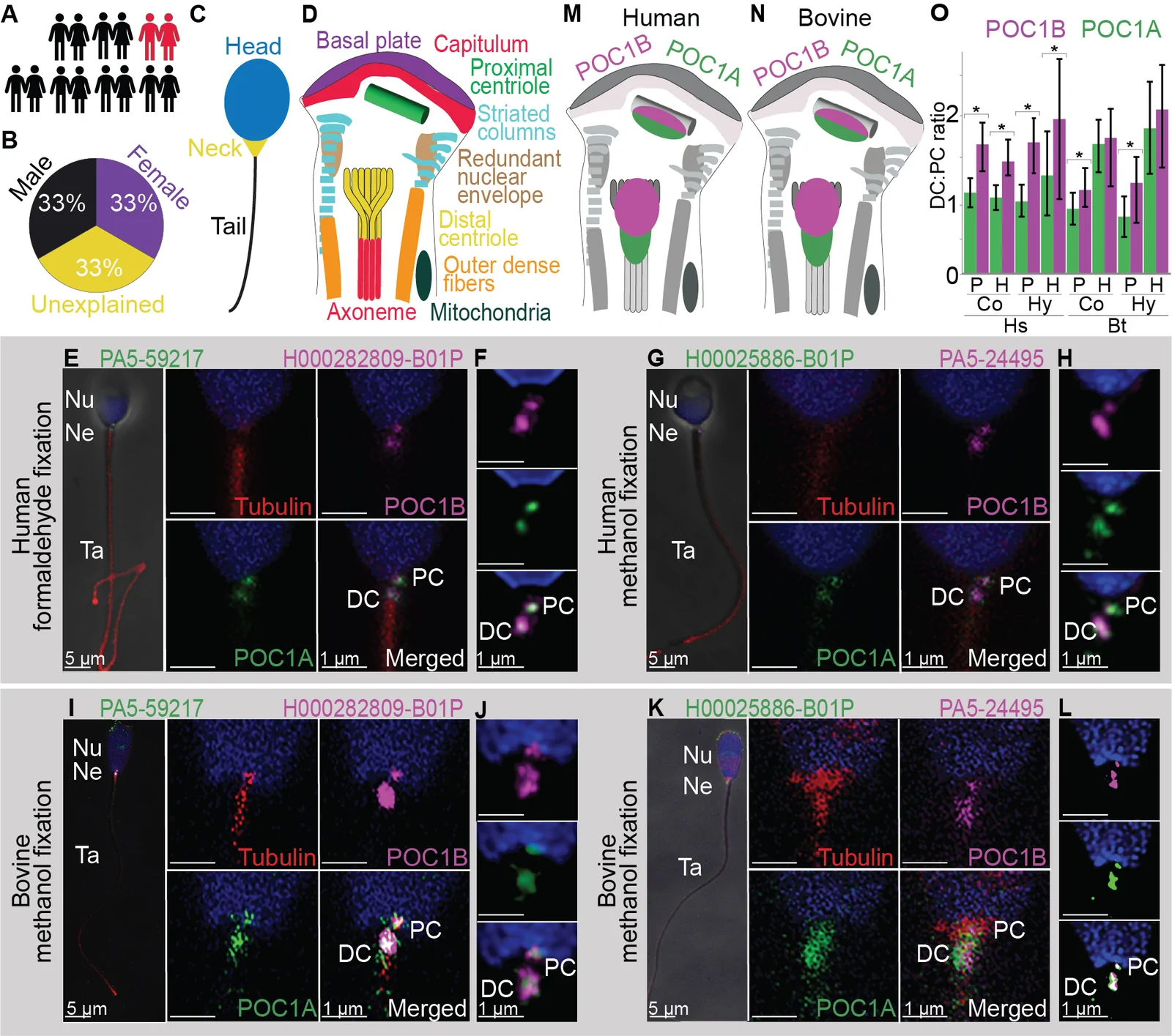



## Description

Infertility is a medical condition affecting 15% of couples globally and can be caused by male and female factors (Cox et al., 2022; Sun et al., 2019) ( **Fig. 1A** ). Up to one-third of infertility couples have unexplained causes (Pandruvada et al., 2021; Raperport et al., 2024; Ray et al., 2012) ( **Fig. 1B** ). One mechanism underlying unexplained infertility is a defect in the spermatozoon neck (Chemes, 2012; Simerly et al., 1997) ( **Fig. 1C, D** ). Recently, abnormalities in spermatozoa centrioles have been reported in human couples with unexplained infertility and in bulls with subfertility (Jaiswal et al., 2022; Turner, Achinger, et al., 2023). To better detect centriole-based infertility, an assay was developed that uses the ratio of centriole protein localization intensities (Turner et al., 2021). This study is part of an ongoing effort to identify centriolar proteins in spermatozoa that may serve as infertility biomarkers (Achinger et al., 2025; Subbiah et al., 2024; Tapia Contreras & Hoyer-Fender, 2021; Turner, Caswell, et al., 2023).

Mammalian spermatozoa consist of a head containing genetic material, a neck with a specialized centrosome, and a tail for motility (Mortimer, 2018) ( **Fig. 1C** ). The centrosome is a microtubule-organizing center that supports cell division and ciliogenesis (Breslow & Holland, 2019). The spermatozoon centrosome undergoes remodeling, resulting in a barrel-shaped proximal centriole (PC), a centriole with an atypical structure and composition, called the distal centriole (DC) (Fishman et al., 2018) ( **Fig. 1D** ). However, the precise structure, composition, and function of the DC remain unclear (Avidor-Reiss et al., 2019).

Proteomic studies identified over 450 potential centriolar proteins with implications for sperm fertility (Alves-Cruzeiro et al., 2014; Amaral et al., 2013; Baker et al., 2013; Baker et al., 2007; Khanal et al., 2024; Wang et al., 2013). However, the localization of only a few of them has been studied in spermatozoa (Achinger et al., 2025; Amargant et al., 2021; Buglak et al., 2024; Firat-Karalar et al., 2014; Galletta et al., 2020; Goto et al., 2010; Subbiah et al., 2024; Takeda et al., 2025; Tapia Contreras & Hoyer-Fender, 2019; Turner, Caswell, et al., 2023; Turner et al., 2022). One such underexplored protein is the Proteome of Centriole Protein 1A (POC1A, aka WDR51A). POC1A dimerizes with the Proteome of Centriole Protein 1B (POC1B) in somatic cells (Sala et al., 2024). POC1B also localizes to spermatozoon centrioles (Fishman et al., 2018). Therefore, we hypothesize that POC1A resides in the spermatozoon neck and colocalizes with POC1B. Mutations in POC1B lead to retinal ciliopathy (Beck et al., 2014) and infertility due to abnormal sperm morphology (Hua et al., 2023) **.** Mutations in POC1A are associated with short stature, onychodysplasia, facial dysmorphia, and hypotrichosis, collectively known as SOFT syndrome (Sarig et al., 2012), as well as Sertoli cell dysfunction (Geister et al., 2015) **.**

To determine the location of proteins in the spermatozoon neck, we used DAPI stain to label the head, an anti-tubulin antibody to label the tail, and an anti-POC1B antibody to label the PC and DC. We used two different POC1A antibodies targeting the human ortholog: H00025886-B01P and PA5-59217. The H00025886-B01P antibody is a polyclonal mouse antibody raised against the full-length amino acid sequence (amino acids 1-364) of isoform AAH07417. The PA5-59217 antibody is a polyclonal rabbit antibody raised against amino acids 262-365 of POC1A.

We studied POC1A localization in humans and bovines. We fixed spermatozoa samples with either methanol or formaldehyde; preliminary results on human spermatozoa showed clearer imaging with PA5-59217 anti-POC1A antibodies after formaldehyde fixation and with H00025886-B01P anti-POC1A antibodies after methanol fixation. Results on bovine spermatozoa showed clearer labeling with methanol fixation using both anti-POC1A antibodies compared to formaldehyde fixation.

We used two POC1B antibodies: PA5-24495 and H000282809-B01P. The H000282809-B01P antibody is a polyclonal mouse antibody raised against the full-length human POC1B protein. The PA5-24495 antibody is a polyclonal rabbit antibody raised against a synthetic peptide from the C-terminal region of human POC1B (amino acids 321-350).

As expected from previous studies, anti-POC1B antibodies consistently labeled both the PC and DC in human and bovine spermatozoa. In human spermatozoa, the formaldehyde-fixed H000282809-B01P anti-POC1B labeled the PC 94% (34/36) and the DC 94% (34/36) of the time. The methanol-fixed PA5-24495 anti-POC1B labeled the PC 94% (33/35) and the DC 97% (34/35) of the time. In bovine spermatozoa, the methanol-fixed H000282809-B01P anti-POC1B labeled the PC 93% (28/30) and the DC 96% (29/30) of the time. The methanol-fixed PA5-24495 anti-POC1B labeled the PC 93% (38/41) and the DC 95% (39/41) of the time.

As expected, we found that human spermatozoa POC1B labeling increased by 1.46±0.26-fold in the DC relative to the PC with PA5-24495 anti-POC1B and by 1.65±0.35-fold with H000282809-B01P anti-POC1B. Bovine spermatozoa POC1B labeling increased by 1.75±0.56-fold in the DC relative to the PC using PA5-24495 anti-POC1B, and by 1.17±0.24-fold using H000282809-B01P anti-POC1B. As hypothesized, we found that POC1A antibodies label the centrioles in human spermatozoa. PA5-59217 anti-POC1A antibody labeled the PC in 94% (34/36) of spermatozoa and the DC in 94% (34/36) of spermatozoa. Similarly, the H00025886-B01P anti-POC1A antibody labeled the PC in 94% (33/35) of spermatozoa and the DC in 94% (33/35) of spermatozoa.

Unlike POC1B, the POC1A labeling intensity, measured by confocal microscopy, was similar between the PC and DC, with a ratio of 1.17±0.21 for PA5-59217 anti-POC1A ( **Fig. 1E- F, O** ), and a ratio of 1.10±0.18 for H00025886-B01P anti-POC1A ( **Fig. 1G- H, O** ). The difference in POC1A and POC1B ratios between the two centrioles is statistically significant (P=4E-10, N=34 for PA5-59217 anti-POC1A and H000282809-B01P anti-POC1B, and P=1E-10, N=31 for H00025886-B01P anti-POC1A and PA5-24495 anti-POC1B, Paired T-Test).

Similarly, the POC1A labeling intensity, measured by HyVolution microscopy, was similar between the PC and DC, with a ratio of 1.02±0.25 for PA5-59217 anti-POC1A ( **Fig. 1E- F, O** ), and a ratio of 1.31±0.56 for H00025886-B01P anti-POC1A ( **Fig. 1G- H, O** ). The difference in POC1A and POC1B ratios between the two centrioles is statistically significant (P=3E-6, N=17 for PA5-59217 anti-POC1A and H000282809-B01P anti-POC1B, and P=0.01, N=17 for H00025886-B01P anti-POC1A and PA5-24495 anti-POC1B, Paired T-Test).

Using PA5-59217 anti-POC1A and H000282809-B01P anti-POC1B antibodies, confocal microscopy results showed a high level of colocalization between POC1A and POC1B in both the PC (Fisher Z=0.84±0.17) and DC (Fisher Z=0.89±0.12) in confocal images. A Paired T-Test indicated a small but statistically significant difference in colocalization levels (P=0.04, D=0.35, N=34) ( **Fig. 1O** ). Similarly, a high level of colocalization was observed with the H00025886-B01P anti-POC1A and PA5-24495 anti-POC1B antibodies (PC: Fisher Z=0.77±0.20; DC: Fisher Z=0.75±0.15). A Paired T-Test of these colocalization levels revealed no significant statistical difference (P=0.52, N=31) ( **Fig. 1O** ).

Similarly, HyVolution images using PA5-59217 anti-POC1A and H000282809-B01P anti-POC1B antibodies showed high colocalization in both the PC and DC. The PC showed a Fisher Z value of 1.05±0.31. The DC showed a Fisher Z value of 1.19±0.24. A Paired T-Test comparing PC and DC Fisher Z values showed no significant difference between them (P=0.14, N=17).

Colocalization analysis using H00025886-B01P anti-PC1A and PA5-24495 anti-POC1B antibodies showed high overlap in signals in both the PC and DC of human spermatozoa in HyVolution images. The PC showed a Fisher Z value of 1.06±0.40. The DC showed a Fisher Z value of 1.08±0.23. A Paired T-Test comparing PC and DC Fisher Z values showed no significant difference between them (P=0.82, N=17).

As hypothesized, we found that POC1A antibodies label the centrioles of bovine spermatozoa. The PA5-59217 anti-POC1A antibody detected the PC in 97% (29/30) of spermatozoa and the DC in 93% (28/30). Similarly, the H00025886-B01P anti-POC1A antibody labeled the PC in 93% (38/41) of spermatozoa and the DC in 95% (39/41).

In confocal imaging of bovine spermatozoa, the PA5-59217 anti-POC1A antibody showed a comparable labeling of the DC and PC, with a ratio of 0.93±0.19 ( **Fig. 1I- J, O** ). Additionally, in bovine spermatozoa, as with POC1B, the POC1A labeling intensity in confocal microscopy was increased in the DC compared to the PC when stained with the H00025886-B01P anti-POC1A antibody, at a ratio of 1.70±0.38-fold ( **Fig. 1K- L, O** ). The difference in POC1A and POC1B ratios between the two centrioles was statistically significant for the PA5-59217 anti-POC1A and H000282809-B01P anti-POC1B antibody pair (P=2E-8, N=31), but not for the H00025886-B01P anti-POC1A and PA5-24495 anti-POC1B antibody pair (P=0.51, N=41; paired T-test).

Unlike POC1B, the labeling intensity of POC1A, measured by HyVolution microscopy, was similar between the PC and DC, with a ratio of 0.82±0.29 for PA5-59217 anti-POC1A ( **Fig. 1E- F, O** ), and a ratio of 1.89±0.67 for H00025886-B01P anti-POC1A ( **Fig. 1G- H, O** ).

The difference in POC1A and POC1B ratios between the two centrioles was statistically significant for the PA5-59217 anti-POC1A and H000282809-B01P anti-POC1B antibody pair (P=0.012, N=13), but not for the H00025886-B01P anti-POC1A and PA5-24495 anti-POC1B antibody pair (P=0.30, N=15; paired T-test).

Colocalization analysis using PA5-59217 anti-POC1A and H000282809-B01P anti-POC1B antibodies showed moderate overlap in signals in both the PC (Fisher Z=0.35±0.09) and the DC (Fisher Z=0.37 ±0.08) in confocal images. A two-proportion T-test indicated no significant difference between the two proportions (P=0.41, N=31). Similarly, confocal images of the H00025886-B01P anti-POC1A and PA5-24495 anti-POC1B antibodies revealed similar moderate colocalization in both the PC (Fisher Z=0.48±0.17) and the DC (Fisher Z=0.48±0.13), with no significant difference between them (P=0.84, N=41, paired T-test comparing H00025886-B01P anti-POC1A with PA5-24495 anti-POC1B).

Colocalization analysis using PA5-59217 anti-POC1A and H000282809-B01P anti-POC1B antibodies showed moderate overlap in signals in both the PC and DC of bovine spermatozoa in HyVolution images. The PC showed a Fisher Z value of 0.41± 0.15. The DC showed a Fisher Z value of 0.77±0.25. A T-test comparing PC and DC Fisher's Z values showed a significant difference between them (P=8E-5, D=1.72, N=13).

HyVolution images using H00025886-B01P anti-POC1A and PA5-24495 anti-POC1B antibodies showed high overlap in both the PC and DC. The PC showed a Fisher Z value of 0.86±0.29. The DC showed a Fisher Z value of 0.77±0.24. A T-test comparing PC and DC Fisher Z values showed no significant difference between them (P=0.31, N=15). Control staining was performed using only secondary antibodies for human and bovine spermatozoa. No signal was detected in the PC or DC ( **Extended data Fig. 1** ).

Overall, POC1A colocalized with POC1B at the PC and DC in both human and bovine spermatozoa using two independently generated antibodies ( **Fig. 1L** ). Since POC1A and B are paralogs with 60% identity in humans and 62% identity in bovine, it is possible that the POC1A antibody recognizes POC1B. We think this is unlikely because the POC1A and B antibodies have different staining patterns. It is also possible that POC1A antibodies recognize other proteins. We also think this is unlikely because the independently generated POC1A antibodies exhibit a similar staining pattern. Therefore, we propose that POC1A can serve as an additional biomarker for centriole quality.

Interestingly, the distribution of POC1A in the PC and DC differed from that of POC1B in three of the four cases we studied (Human and Bovine PA5-59217, as well as Human H00025886-B01P, but not Bovine H00025886-B01P), suggesting that the POC1A:POC1B ratio varies between these regions. POC1A is a component of the centriolar helical inner scaffold, together with POC1B, FAM1611A, POC5, CCDC15, WDR90, and Centrin 1/2 (Arslanhan et al., 2023; Le Guennec et al., 2020; Sala et al., 2024; Steib et al., 2020). This suggests that the proteins of the centriolar helical inner scaffold are present in different quantities in the spermatozoon centrioles, possibly reflecting distinct functions.

## Methods

**Spermatozoa preparation**

A previously used protocol was used to prepare human (Jaiswal et al., 2022) and bovine (Turner et al., 2022) spermatozoa. Here we provide a brief description.

**Human** : Samples from the Reproductive Subject Registry and Sample Repository (RSRSR) at the University of Michigan (Schon et al., 2021) were thawed and separated into pellet and interface fractions. 1 mL of PureSperm® 80% (Nidacon, PS80-100) and 1 mL of PureSperm® 40% (Nidacon, PS40-100) were pipetted sequentially into a 15 mL conical tube, followed by a semen sample. The tube was centrifuged at 400g for 20 minutes, and the supernatant was discarded. The resulting pellet was washed with 2 mL of PureSperm® wash media (Nidacon, PSW-100), then centrifuged at 250g for 8 minutes. The supernatant was discarded again, and the pellet was resuspended in 100 μL of Medium 199 (Sigma-Aldrich, M7528). 10 μL were then pipetted onto a glass slide, covered with a coverslip, and flash-frozen in liquid nitrogen.

**Bovine** : Bovine spermatozoa straws were retrieved from liquid nitrogen storage and thawed in a 37°C water bath. The thawed contents were transferred to a 15 mL conical tube containing 2 mL of PureSperm® Wash medium (Nidacon, PSW-100). Samples were centrifuged at 250g for 8 minutes. The resulting pellet was resuspended in 100 μL of mKRH buffer; 50 μL was pipetted onto a glass slide, covered with a coverslip, and immediately frozen in liquid nitrogen for storage until further use.

**Spermatozoa staining**

**Human:** Slides containing human spermatozoa were removed from liquid nitrogen storage, and the coverslips were pried off using forceps. The slides were fixed in a Coplin jar containing either room-temperature (20–25°C) 3.7% formaldehyde for 10 minutes or ice-cold (−4°C) methanol for 3 minutes. After fixation, the slides were washed in room-temperature 1× PBS for 10 minutes, then permeabilized in 1× PBS containing 0.3% Triton X-100 (PBST) for 1 hour at room temperature. The slides were then blocked in PBST with 5% bovine serum albumin (PBSTb) for 1 hour at room temperature. Following blocking, they were transferred to a humidity chamber, incubated with 100 µL of PBSTb containing diluted primary antibodies, covered with Parafilm, and left overnight (≥16 hours) at 4°C. The slides were washed three times for 5 minutes each in PBST. Secondary antibodies and Hoechst, diluted in PBSTb (100 µL), were added; the slides were covered with Parafilm and incubated at room temperature for 4 hours. Afterward, they were washed three times for 5 minutes each in PBST, followed by three more washes for 5 minutes each in PBS. A drop of Fluoroshield mounting medium was applied, the coverslip was placed on top, the assembly was sealed with nail polish, and it was stored at 4°C until imaging.

**Bovine:** Slides containing bovine spermatozoa were removed from liquid nitrogen storage, and the coverslips were pried off using forceps. Slides were fixed in a Coplin jar containing ice-cold (−4°C) methanol for 3 minutes. After fixation, slides were washed in room-temperature 1× PBS for up to 1 hour and then permeabilized in PBST for 1 hour at room temperature. Slides were blocked in PBSTb for 30 minutes at room temperature. After blocking, slides were transferred to a humidity chamber, incubated with primary antibodies diluted in PBSTb, covered with Parafilm, and incubated for 36 hours at 4°C. Slides were washed three times for 5 minutes each in PBST. Secondary antibodies and Hoechst, both diluted in PBSTb, were then applied, and slides were incubated at room temperature for 2 hours. Slides were washed three times for 5 minutes each in PBST, followed by three washes for 5 minutes each in PBS. A drop of mounting medium was added, covered with a coverslip, sealed with nail polish, and stored at 4°C until imaging.

**Spermatozoa visualization**

**Confocal:**

Slides were visualized using a Leica SP8 confocal microscope in Bright-R mode with an HC PL APO CS2 63x/1.40 OIL lens, 100% gain, 1024 x 1024-pixel format, 3x zoom, line averaging of 3, and frame accumulation of 2. Human samples used a frame accumulation of 3, while bovine samples used 2. Three sequences collected fluorescent signals. In sequence one, DNA and phase-like images were made using a 405 nm laser. Emissions were stimulated with the UV laser set to 0.01%, detected by the HyD1 detector between 410 and 478 nm, and then color-coded blue. In sequence two, secondary antibodies raised against primary antibodies in mice were excited with a 488 nm laser at 3% in human spermatozoa and 2.5% in bovine spermatozoa. Emissions were captured with a HyD3 detector between 483 and 551 nm and color-coded green. Secondary antibodies targeting primary antibodies made in rabbits were stimulated with a 647 nm laser at 1.5% for human spermatozoa and 1% for bovine spermatozoa. Emissions were captured with a HyD4 detector between 638 and 718 nm, color-coded magenta. In sequence three, tubulin antibodies were excited with a 561 nm laser at 0.75% in both human and bovine spermatozoa. Tubulin antibody emissions were captured by HyD3 detectors between 566 and 623 nm, color-coded red. Ten to twenty Z-sections of 0.3µm thickness were collected from the bottom to the top of the spermatozoa. During figure creation, POC1A was shown in green and POC1B in magenta.

**HyVolution:**

When imaging both human and bovine spermatozoa, the same conditions as above were used, except that a 6x zoom factor was employed and the gain was set between 10% and 40%. Images were deconvolved using HyVolution II (Leica Microsystems). The SVI Huygens Essential program was used with the maximum resolution strategy.

**Image preparation and analysis**

**Confocal:** TIF images were created using confocal microscopy and imported into Adobe Photoshop. Saturation levels were adjusted to enhance staining visibility, and images were rotated to align the spermatozoa so that the neck was straight, with the PC on the right. These images were then placed into a panel in Adobe Illustrator, along with corresponding labels and scale bars. Images of the spermatozoa head, neck, and centrioles were cropped to 75 x 75 pixels at 300 dpi and resized to 1 in x 1 in. Additionally, images of the spermatozoa head, neck, centrioles, and tail were cropped to 150 x 450 pixels at 300 dpi and resized to 0.667 in x 2 in.

**HyVolution:** TIF images were created as described above at 300 dpi and 75 x 75 pixels. Images were adjusted to 0.667 in x 0.667 in.

**Statistics**

To gather information for statistical analysis of human and bovine spermatozoa, LAS X software was used in “Quantify Mode.” Z-stack images of spermatozoa were projected, with the projection set to “maximum projection.” Subsequently, a 1 µm × 0.75 µm region of interest was drawn around a centriole. Mean intensities of POC1A and POC1B within the centriole were recorded for subsequent statistical analysis.

A colocalization report was then generated for the centrioles, recording the Pearson correlation coefficient between POC1A and POC1B, with the POC1A and POC1B channels set to a 30% threshold using the confocal pictures. The Pearson correlation value was then converted to a Fisher Z value using Microsoft Excel’s Fisher command (Achinger et al., 2025; Sánchez-Meca et al., 2013). Only samples containing a visible PC and DC were quantified. Because inclusion was based on PC/DC visibility and antibody-pair-specific image quality, the final number of sperm analyzed differed between antibody-pair conditions in bovine spermatozoa, with 41 sperm quantified for H00025886-B01P anti-POC1A antibody and PA5-24495 anti-POC1B antibody, and 31 sperm quantified for PA5-59217 anti-POC1A antibody and H000282809-B01P anti-POC1B antibody. The mean intensities of POC1A and POC1B were then used to calculate the DC:PC enrichment ratio for each. The method was then used to gather statistics using HyVolution images.

## Reagents

**Table:** 

**Antibody Name**	**Referred to as:**	**Host Species**	**Manufacturer**	**Product Number**	**Lot Number**	**Human Fixation Method**	**Human Dilution**	**Bovine Fixation Method**	**Bovine Dilution**
**WDR51A Polyclonal Antibody, MaxPab™**	H00025886-B01P anti-POC1A	Mouse	Abnova	H00025886-B01P	LA281	Methanol	1:75	Methanol	1:50
**POC1A Polyclonal**	PA5-59217 anti-POC1A	Rabbit	ThermoFisher (Invitrogen)	PA5-59217	ZL4563696B	Formaldehyde	1:75	Methanol	1:50
**WDR51B purified MaxPab polyclonal**	H000282809-B01P anti-POC1B	Mouse	Abnova	H000282809-B01P	NB131	Formaldehyde	1:100	Methanol	1:100
**POC1B Polyclonal Antibody**	PA5-24495 anti-POC1B	Rabbit	ThermoFisher (Invitrogen)	PA5-24495	79475494	Methanol	1:100	Methanol	1:100
Anti-alpha/beta tubulin: polyclonal		Sheep	Cytoskeleton Inc.	ATN02	105	Formaldehyde/ Methanol	1:600	Methanol	1:600
**Mouse IgG (H+L) Cross-Adsorbed Antibody, DyLight™ 488**		Donkey	ThermoFisher (Invitrogen)	SA5-10166	YD3896532	Formaldehyde/ Methanol	1:500	Methanol	1:400
**Rabbit IgG (H+L) Cross-Adsorbed Antibody, DyLight™ 650**		Donkey	ThermoFisher (Invitrogen)	SA5-10041	XJ3722883	Formaldehyde/ Methanol	1:500	Methanol	1:400
**Sheep IgG (H+L) Cross-Adsorbed Antibody, Alexa Fluor™ 555**		Donkey	ThermoFisher (Invitrogen)	A21436	2420712	Formaldehyde/ Methanol	1:1000	Methanol	1:1000
**Hoechst 33342**			ThermoFisher (Invitrogen)	H1399		Formaldehyde/ Methanol	1:2000	Methanol	1:2000

The primary antibody staining of H000282809-B01P anti-POC1B and PA5-24495 anti-POC1B was previously validated (Turner et al., 2022).

The primary antibody staining of POC1A was validated using a secondary control; no staining was observed in the absence of the primary antibody ( **see Extended data Figure** ).

Solutions:

Washing solution: Phosphate-buffered Saline (PBS)

Permeabilization Buffer (PBST): is made of PBS with 0.3% Triton X-100 (Sigma-Aldrich, X100-500ML)

Blocking Solution (PBSTb): is made of PBST with 1% or 5% BSA (Bovine Serum Albumin: Roche, 10735086001)

Hoechst Stain: Thermo Fisher Scientific, H1399 (10 mg/mL)

Fixation Media: Methanol (-20˚C) (Fisher Chemical, A412P-4), 37% Formaldehyde (Sigma-Aldrich, 252549- 100ML, diluted to 3.7%)

Mounting Media: Fluoroshield with DAPI (Sigma-Aldrich, F6057-20ML)

PureSperm® 80% (Nidacon, PS80-100)

PureSperm® 40% (Nidacon, PS40-100)

PureSperm® wash media (Nidacon, PSW-100)

mKRH Buffer: 39.6 mL Milli-Q Water, 0.220 g NaCl, 0.012 g MgSO4 7H2O, 0.006 g KH2PO4, 0.014 g KCl, 0.239 g Na HEPES, 0.199 g Glucose, 400 µL 100× penicillin-streptomycin solution.

**Samples:**

Bovine spermatozoa were provided by Select Sires, Inc.

Human spermatozoa were considered to be fertile samples from the Reproductive Subject Registry and Sample Repository (RSRSR) (Schon et al., 2021) at the University of Michigan (UM IRB#HUM00125627). The Institutional Review Board (IRB) at the University of Toledo approved this study (UT IRB#300364; initial approval 10/11/2019; PI: Tomer Avidor-Reiss).

**Materials**

Clear nail polish (Electron Microscopy Sciences, 72180)

Micro glass coverslips (VWR, 48366-205)

Parafilm Wax (VWR, 52858-032)

Glass slides (Azer Scientific, 2752511)

Glass Coplin jars (Research Products International, 144206)

## Data Availability

Description: Extended data Figure and Legend. Resource Type: Text. DOI: https://doi.org/10.22002/kbywq-ctz95
